# Supplementary material for: Barriers to USMLE Step-1 accommodations: Students with Type 1 Diabetes
Source: PLoS One. 2024 Jun 18;19(6):e0304784. doi: 10.1371/journal.pone.0304784 (PMC11185460; doi:10.1371/journal.pone.0304784)
Supplement: S1 Appendix — (DOCX) [file pone.0304784.s001.docx]

**Appendix 1.** Survey instrument

**Section 1: History of Diabetes and Accommodations**

1. Age of diagnosis with Type 1 Diabetes? (fill in)
2. Have you received accommodations in the past? (MCAT, ACT/SAT) (yes/no)
3. I received accommodations on the following standardized exams (please specify the accommodations): (fill in)
4. What calendar month and year did you take the USMLE Step-1 Exam? (fill in)

**Section 2: Applying for Step-1 Accommodations**

1. Did you request accommodations for the USMLE Step-1 Exam? (yes/no) (if no, answer 1a); If yes, go on to 2.
2. Why didn’t you apply for accommodations for the USMLE Step-1 Exam? (fill in)
3. What accommodations did you request for the USMLE Step-1 Exam? (fill in)
4. After your initial application submission, did the USMLE contact you to submit any additional documentation? (yes/no) (if no, answer 3a); If yes, go on to 4
   1. What additional documentation did you have to submit? (fill in)
5. What accommodations did you receive for the USMLE Step-1 Exam? (fill in)
6. After your initial application submission, how long did it take to receive the letter approving/denying your accommodations? (fill in)
7. Did you choose to go through an appeals process? (yes/no) (if no, answer 6a); If yes, go on to 7
   1. Why did you choose NOT to go through the appeals process? (fill in)
8. What was the outcome of the appeals process? (fill in)
9. On a scale of 1 to 7, overall what level of stress was associated with the process of requesting accommodations for USMLE Step-1 Exam? (7 point Likert scale)
10. How did USMLE’s process of applying for accommodations make you feel? (fill in)

**Section 3: Exam Day**

1. What was your experience the day(s) of the exam with/without the accommodations received? (fill in)
2. Share anything you’d like us to know about your experience with the process of applying for accommodations. (fill in)
3. Did you pass the Step-1 exam on the first attempt? (fill in)
