# Supplementary material for: Barriers to USMLE Step-1 accommodations: Students with Type 1 Diabetes
Source: PLoS One. 2024 Jun 18;19(6):e0304784. doi: 10.1371/journal.pone.0304784 (PMC11185460; doi:10.1371/journal.pone.0304784)
Supplement: Attachment — Submitted filename: Response to Reviewers.docx [file pone.0304784.s002.docx]

Dr. Mukhtiar Baig, Ph.D.

Academic Editor

Barriers to USMLE Step-1 Accommodations: Students with Type 1 Diabetes - PLOS ONE

PONE-D-24-10049

We are grateful to the reviewers for their thoughtful feedback regarding our manuscript. We have edited the conclusion to address limitations and biases. Please see below for point-by-point responses to comments and the updated manuscript. Please let us know if you have additional suggestions to improve our manuscript. We appreciate this opportunity!

Emily Serata, BS

Dr. Emily Noonan, PhD

Dr. Kristina Petersen, PhD

1. Please ensure that your manuscript meets PLOS ONE's style requirements, including those for file naming. The PLOS ONE style templates can be found at 
<https://journals.plos.org/plosone/s/file?id=wjVg/PLOSOne_formatting_sample_main_body.pdf> and 
<https://journals.plos.org/plosone/s/file?id=ba62/PLOSOne_formatting_sample_title_authors_affiliations.pdf>

**Response:** Table captions have been moved so they appear before the table. Citations have been changed from superscript to brackets. Spacing has been edited.

2. Thank you for uploading your study's underlying data set. Unfortunately, the repository you have noted in your Data Availability statement does not qualify as an acceptable data repository according to PLOS's standards.

At this time, please upload the minimal data set necessary to replicate your study's findings to a stable, public repository (such as figshare or Dryad) and provide us with the relevant URLs, DOIs, or accession numbers that may be used to access these data. For a list of recommended repositories and additional information on PLOS standards for data deposition, please see <https://journals.plos.org/plosone/s/recommended-repositories>.

**Response:** The data set has been uploaded to figshare as follows: <https://figshare.com/s/97e926743504b2990045>.

3. Please upload a copy of Supporting Information Figure/Table/etc. S1 Appendix which you refer to in your text on page 29.

**Response:** The data where these quotes come from are uploaded in the figshare as follows: <https://figshare.com/s/97e926743504b2990045>.

4. Please review your reference list to ensure that it is complete and correct. If you have cited papers that have been retracted, please include the rationale for doing so in the manuscript text, or remove these references and replace them with relevant current references. Any changes to the reference list should be mentioned in the rebuttal letter that accompanies your revised manuscript. If you need to cite a retracted article, indicate the article’s retracted status in the References list and include a citation and full reference for the retraction notice.

**Response:** No references have been retracted.

Reviewer's Responses to Questions

**Comments to the Author**

1. Is the manuscript technically sound, and do the data support the conclusions?

The manuscript must describe a technically sound piece of scientific research with data that supports the conclusions. Experiments must have been conducted rigorously, with appropriate controls, replication, and sample sizes. The conclusions must be drawn appropriately based on the data presented.

Reviewer #1: Yes

Reviewer #2: Partly

**Response:** 21 medical students at LCME-accredited MD programs identified as having T1D and indicated interest in participating in the survey were sent anonymous surveys. 16 of these participants responded. In reference to the small sample size, there has been a calculated 5.7% prevalence, according to the CDC, of the general population with Type 1 Diabetes and a much smaller percentage of medical students with Type 1 Diabetes. Additionally, we only accounted for MD students in LCME-accredited programs who had taken Step 1 within the last 3 years. Due to the extremely limited number of medical students with Type 1 Diabetes who had taken the USMLE Step 1 Exam within the last 3 years, we feel our sample size (N=16) represents enough students to gather qualitative data (similar to Stergiopolous et al., 2018 where their sample size was n=10 for a qualitative study about students with disabilities).

2. Has the statistical analysis been performed appropriately and rigorously?

Reviewer #1: I Don't Know

Reviewer #2: Yes

**Response:** Due to the nature of the results being qualitative and the small sample size, thematic analysis was used to find themes and divide the comments into four main categories: general satisfaction, anger, frustration, stress as seen in **Table 2**. This was accomplished by splitting the comments into separate categories depending on their responses to Q12-Q14 and having revisions by all authors to ensure lack of misinterpretation. Responses were split into groups as seen in **Figure 1** based first on their response to Q3. For those who did apply to Step 1 accommodations, responses to Q5 and Q6 were compared to understand if they received what they had requested. For both groups to understand if barriers were faced on test day, Q13-Q14 were assessed.

3. Have the authors made all data underlying the findings in their manuscript fully available?

The [PLOS Data policy](https://nam11.safelinks.protection.outlook.com/?url=http%3A%2F%2Fwww.plosone.org%2Fstatic%2Fpolicies.action%23sharing&data=05%7C02%7Cserata64%40studentsrowan.mail.onmicrosoft.com%7C29817cbe9ac04b57737408dc6d9441e2%7Ceda8e9bc72cf449ca4e6725e6c6bd0d8%7C0%7C0%7C638505730755590994%7CUnknown%7CTWFpbGZsb3d8eyJWIjoiMC4wLjAwMDAiLCJQIjoiV2luMzIiLCJBTiI6Ik1haWwiLCJXVCI6Mn0%3D%7C0%7C%7C%7C&sdata=nDQIHT%2FHpCs63DWal9weMvjG1fkv1j7NPXKY%2FkmmwZ4%3D&reserved=0) requires authors to make all data underlying the findings described in their manuscript fully available without restriction, with rare exception (please refer to the Data Availability Statement in the manuscript PDF file). The data should be provided as part of the manuscript or its supporting information, or deposited to a public repository. For example, in addition to summary statistics, the data points behind means, medians and variance measures should be available. If there are restrictions on publicly sharing data—e.g. participant privacy or use of data from a third party—those must be specified.

Reviewer #1: Yes

Reviewer #2: Yes

**Response:** All data has been made available on figshare as follows: <https://figshare.com/s/97e926743504b2990045>.

4. Is the manuscript presented in an intelligible fashion and written in standard English?

PLOS ONE does not copyedit accepted manuscripts, so the language in submitted articles must be clear, correct, and unambiguous. Any typographical or grammatical errors should be corrected at revision, so please note any specific errors here.

Reviewer #1: Yes

Reviewer #2: Yes

**Response:** N/A

5. Review Comments to the Author

Please use the space provided to explain your answers to the questions above. You may also include additional comments for the author, including concerns about dual publication, research ethics, or publication ethics. (Please upload your review as an attachment if it exceeds 20,000 characters)

Reviewer #1: Dear researcher,

Thank you for submitting your research. I found your work to be impressive and insightful. However, I noticed that the sample size was relatively small, which might limit the scope of the findings. Additionally, it might be worth considering the possibility of recall bias, as the students who participated in the study were asked to recall their experiences several months after taking the USMLE Step 1 test.

Nevertheless, I appreciate how your research is a commendable effort to identify areas for improvement in the USMLE process to better accommodate students with disabilities. Your work has the potential to make a significant impact on the medical education community, and I encourage you to continue your research in this area.

Thank you for your contributions to the field.

Best regards.

**Response:** Thank you for your time and response. We have added a paragraph in the discussion to address the sample size and recall bias:

“…Limitations of this study include the small sample size (N=16) of participants and the niche nature of the disease. Because the prevalence of Type 1 Diabetes is 5.7% and the amount of medical students with reported chronic disability is significantly decreased from the national prevalence, there was a decreased sample of eligible candidates[39]. We additionally only surveyed those who were MD students at LCME-accredited institutions who had taken USMLE Step 1 within the last 3 years, which was another barrier to increasing our sample size. While we limited this time-frame in order to decrease the gap in time since students took the exam, results should be viewed through a cautious lens as recall bias could impact our results. One point to consider is that only one individual in the sample did not pass the USMLE Step 1 Exam, potentially decreasing the effect of negative recall bias in most participants…”

Thank you for the continued consideration of this manuscript and please let us know if there are any further changes to be made.

Reviewer #2: The study has tried to explore negative experiences of a marginalized population of medical students (with type 1 DM) as a result of non-provision of accommodation during USMLE STEP 1 exam. But the sample size is too small, so the results cannot be generalized.
The methodology and statistical analysis are sound for the qualitative design of the study. However, some additional aspects could have been explored like participant's characteristics (e.g. level of stress) that could have contributed to their negative experiences during USMLE exam.
The use of controls in the study would have strengthened the methodology and lead to more meaningful interpretations.
My query to the authors: Is lack of accommodation the only or the major factor for negative experiences of medical students with type 1 DM during USMLE exam? Please elaborate on other factors that could have lead to their ill-health during USMLE exam in the discussion.

**Response:** Thank you for your time and response. You bring up a good point regarding lack of accommodation being the major factor for negative experiences of medical students with Type 1 DM during the USMLE Exam. We felt that by asking students about their history with past accommodations for exams, that we could better understand their history with accommodations compared to what they received on this exam. While lack of accommodations will not directly cause negative impact on their health during the exam, the lack of accommodations (and anticipation/anxiety over what could occur without accommodations leading up to the exam date) induces stress due to lack of time and space to care for themselves when ill-health outcomes arise. As we describe in our manuscript:

“…People with varying disabilities require Step-1 accommodations, including those who have a primary diagnosis of T1D (9.5% prevalence in U.S.)[19]. T1D is defined as a chronic autoimmune disease characterized by unstable glucose levels due to insulin deficiency leading to events of hyper/hypoglycemia and is protected under the ADA[20, 21]. Hyperglycemia occurs when glucose levels are high, resulting in extreme thirst, unintentional weight loss, loss of energy, nausea, trouble seeing, confusion, drowsiness, and poor concentration[22]. Conversely, hypoglycemia occurs when glucose levels are low, causing tachycardia, sweating, nervousness, nausea, difficulty concentrating, and confusion[22]^,^[23]. Severe hypoglycemia may cause loss of consciousness, seizure, and coma; such episodes are life-threatening, and require immediate medical attention[23, 24]. Episodes of both hyper- and hypo-glycemia may occur with rapid onset; these incidents are unpredictable and interfere with activities of daily living, employment, and schooling[25]. Therefore, accommodations in the workplace and educational settings are necessary for individuals with T1D to allow them equal opportunities and access…”

Therefore, we feel that accommodations to care for hyper- and hypo- glycemic episodes are necessary for students with T1D to fully demonstrate their abilities. In terms of stress levels, taking this exam is inherently a stressful experience for any student, but most students do not have an additional layer of health concern to consider that could adversely impact their cognition and full ability to perform on the 7-hour exam. All students who had difficulties with their test-taking day specifically described hyper/hypo-glycemic episodes or issues with their diabetes-related technology, such as an insulin pump.

We stated this information in the results section when citing patients’ specific experiences with adverse events on test-day. We also added 2 sentences addressing this in the discussion:

“A combination of diabetes-related barriers, such as hypo/hyper-glycemia and insulin pump malfunctions, as well as associated stress due to situations like the continual insulin pump alarm seem to both have contributed to poor experiences on test-day. While additional factors, such as stress and anxiety, may have played a role in the negative experiences as has been reported as being the case for many students who take this exam[39-42], participants repeatedly cited diabetes-related barriers as their main source of stress on test-day…”

Thank you for sharing your concern about the samples size and lack of a control group.

We also added information to address the small sample size below. We believe the criticism about the lack of a control group is misplaced. The goal of our research was not to study the differences between students with T1D and those without. Rather, our goal was to explore the experiences of students with T1D with the USMLE Step 1 exam. Generally, qualitative research does not include control groups.

“…Limitations of this study include the small sample size (N=16) of participants and the niche nature of the disease. Because the prevalence of Type 1 Diabetes is 5.7% and the amount of medical students with reported chronic disability is significantly decreased from the national prevalence, there was a decreased sample of eligible candidates[39]. We additionally only surveyed those who were MD students at LCME-accredited institutions who had taken USMLE Step 1 within the last 3 years, which was another barrier to increasing our sample size. While we limited this timeframe in order to decrease the lag since the exam and students’ experiential reports, recall bias still stands as a potential to consider in evaluating our results. One point to consider is that only one individual in the sample did not pass the USMLE Step 1 Exam, potentially decreasing the effect of negative recall bias in most participants. Additional limitations include our selection of students via relevant listservs and networking rather than a randomized approach. Future work includes surveying more individuals with specific disability diagnoses regarding barriers they may face in the USMLE accommodations application process…”

Thank you for the continued consideration of this manuscript and please let us know if there are any further changes to be made.
